# Bare metal stent versus paclitaxel eluting stent for intermediate length femoropopliteal arterial lesions (BATTLE trial): study protocol for a randomized controlled trial

**DOI:** 10.1186/1745-6215-15-423

**Published:** 2014-10-30

**Authors:** Yann Gouëffic, Adrien Kaladji, Béatrice Guyomarch, Carine Montagne, Damien Fairier, Simon Gestin, Valéry-Pierre Riche, Pierre Alexandre Vent, Philippe Chaillou, Alain Costargent, Philippe Patra

**Affiliations:** CHU Nantes, l’institut du thorax, service de chirurgie vasculaire, Nantes, F-44000 France; INSERM, U957, Nantes, F-44000 France; Université de Nantes, Nantes, F-44000 France; CHU Nantes, l’institut du thorax, centre d’investigation clinique, Nantes, F-44000 France; CHU Brest, service de médecine interne 1 et de médecine vasculaire, Brest, F-29200 France

**Keywords:** Superficial femoral artery, Bare metal stent, Drug eluting stent, Paclitaxel

## Abstract

**Background:**

Currently, endovascular treatment is indicated to treat femoropopliteal lesions ≤15 cm. However, the Achilles’ heel of femoropopliteal endovascular repair remains restenosis. Paclitaxel eluting stents have shown promising results to prevent restenosis in femoropopliteal lesions compared to percutaneous transluminal angioplasty. A recently released prospective registry using a newer generation of self-expandable nitinol stents (Misago®; Terumo Corp., Tokyo, Japan) supports primary bare metal stenting as a first-line treatment for femoropopliteal lesions. To date, no studies have been designed to compare bare metal stents to paclitaxel eluting stents for the treatment of femoropoliteal lesions. The BATTLE trial was designed to compare paclitaxel eluting stents (Zilver® PTX®) and a last generation bare self-expandable nitinol stents (Misago® RX, Terumo Corp., Tokyo, Japan) in the treatment of intermediate length femoropopliteal lesions (≤14 cm).

**Methods/Design:**

A prospective, randomized (1:1), controlled, multicentric and international study has been designed. One hundred and eighty-six patients fulfilling the inclusion criteria will be randomized to one of the two assessments of endovascular repair to treat *de novo* femoropopliteal lesions ≤14 cm in symptomatic patients (Rutherford 2 to 5): bare stent group and paclitaxel eluting stent group. The primary endpoint is freedom from in-stent restenosis at 1 year defined by a peak systolic velocity index >2.4 (restenosis of >50%) at the target lesion and assessed by duplex scan. Our main objective is to demonstrate the clinical superiority of primary stenting using Zilver® PTX® stent system versus bare metal self-expandable stenting in the treatment of femoropopliteal lesions in patients with symptomatic peripheral arterial disease.

**Discussion:**

This is the first randomized and controlled study to compare the efficacy of bare metal stents and paclitaxel eluting stents for the treatment of femoropopliteal lesions. It may clarify the indication of stent choice for femoropopliteal lesions of intermediate length.

**Trial registration:**

Clinicaltrials.gov identifier: NCT02004951. 3 December 2013.

## Background

In cases of atheromatous lesions of the femoropopliteal segment, open or endovascular procedures could be proposed to revascularize this segment. Over the past years, endovascular procedures have become an important part of treatment in patients with peripheral arterial disease [1]. Indications for endovascular repair of femoropopliteal lesions have been considerably enlarged as shown in the Trans-Atlantic Inter-Society Consensus Document on Management of Peripheral Arterial Disease (TASC) [1]. Enlargement of endovascular therapy indication is based on patient choice for a less invasive technique and evidence-based medicine. Consequently, the TASC classification of lesions has been modified to reflect increased evidence for endovascular treatment of more extensive femoropopliteal lesions, and indications for endovascular repair have been expanded to more severe TASC types. Currently, endovascular treatment is indicated for femoropopliteal lesions ≤15 cm [1]. Few randomized and controlled studies have been performed to compare treatment strategies for femoropopliteal lesions. Indeed, only three randomized studies have showed outcomes in favor of primary stenting [2–4]. The Achilles’ heel of femoropopliteal stenting remains in-stent restenosis. In coronary arteries, drug eluting stents (DES), combining a platform (a bare metal stent) and a drug (cytostatic or cytotoxic drug) with or without a polymer, have shown promising restenosis prevention results [5]. However, limus eluting stent for femoropopliteal lesions failed to show consistent results, as for coronary arteries. Indeed, sirolimus and everolimus drug eluting stents failed to prove significant efficacy over bare metal stents for femoropopliteal lesions [6, 7]. Conversely, in 2011, Dake *et al*. showed that a paclitaxel-eluting stent was superior, in terms of patency and reintervention, to balloon angioplasty with provisional stenting to treat femoropopliteal lesions ≤14 cm [8]. In a second arm of randomization, Dake *et al*. showed that the application of paclitaxel eluting stents reduced restenosis and reinterventions compared to bare metal stent. Although this study demonstrated the superiority of paclitaxel eluting stents over bare metal stents, the study was not designed to compare bare metal stents to DES. Consequently, no current studies have compared bare stents to paclitaxel-eluting stents as a primary objective for the treatment of the femoropopliteal segment.

Recently, Schulte *et al*. published the results of the Misago® 2 trial [9]. The Misago® 2 trial was a prospective, non-randomized multicenter study which evaluated the safety and efficacy of a latest generation nitinol stent with the first rapid-exchange (RX) monorail system (Misago®, Terumo Corp., Tokyo, Japan). Seven hundred and forty-four patients were enrolled in this study and followed up for at least 1 year. During the inclusion period, 750 femoropopliteal lesions were treated with a mean lesion length of 63 mm. The authors showed a promising efficacy of the Misago® RX nitinol stent system with a clinically driven target lesion revascularization (TLR) rate of 89.9% at 1 year compared with 90.5% for the paclitaxel eluting stent group in the Zilver® PTX® randomized study [8]. Moreover, primary patency was recorded in 574 (87.6%) patients evaluated at 1 year post procedure. Although Misago® 2 was designed as a prospective registry without comparison to other therapeutic options, this trial supports primary stenting and the use of the Misago® RX nitinol stent as a first-line treatment for femoropopliteal lesions.

The primary objective of the BATTLE trial is to demonstrate the clinical superiority of primary stenting using the Zilver® PTX® stent system versus a latest generation bare nitinol stent (Misago® RX, Terumo Corp., Tokyo, Japan) in the treatment of intermediate length femoropopliteal lesions in patients with symptomatic peripheral arterial disease (Rutherford 2 to 5).

## Methods

The CONSORT Statement has been used to report the trial’s design, conduct, analysis and interpretation, and to assess the validity of its results [10, 11]. The study has received ethics approval (France: Comité de Protection des Personnes Ouest IV; Switzerland: in process).

### Design of the study

**Figure 1:**
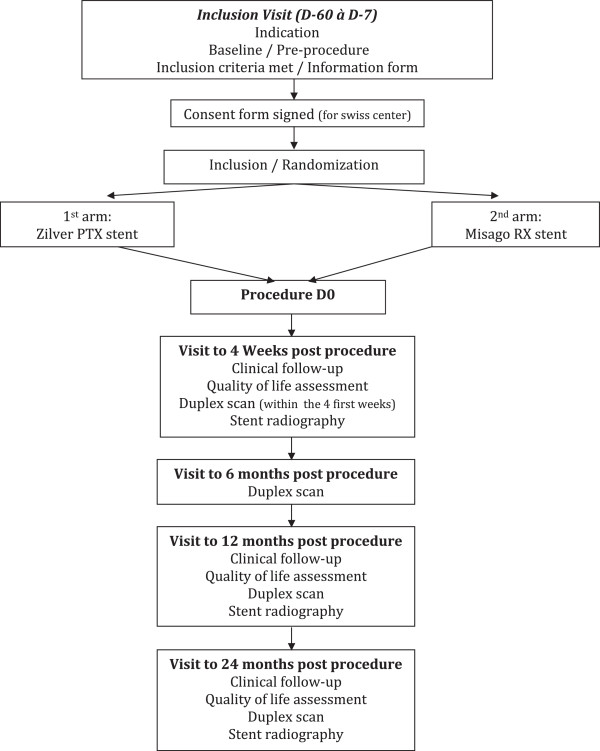
**Flow chart.** Flow diagram of progress through the phases of a parallel-randomized trial of two groups.

The BATTLE clinical investigation is a prospective, randomized (1:1), controlled, multicentric and international trial in France and Switzerland. All randomized patients will be included in the analysis (intent to treat principle). The objective is to demonstrate the clinical superiority of primary stenting using the Zilver® PTX® stent system versus a latest generation bare nitinol stent (Misago® RX, Terumo Corp., Tokyo, Japan) in the treatment of intermediate length femoropopliteal lesions in patients with symptomatic peripheral arterial disease (Rutherford 2 to 5). The expected overall duration of the study is 48 months. The enrollment period is 24 months and the patient follow-up period is 24 months. The study design is summarized in Figure 1.

## Study objectives and endpoints

### Main objective

The main objective of the study is to demonstrate the clinical superiority of primary stenting using the DES (Zilver® PTX®) stent system versus bare metal self-expandable stenting (Misago® RX) in the treatment of intermediate length femoropopliteal lesions in patients with symptomatic peripheral arterial disease (Rutherford 2 to 5).

### Primary endpoint

The study is powered based on the primary endpoint of freedom from in-stent restenosis at 1 year, assessed by duplex scan. Each examination comprises measurements of the maximum peak systolic velocity (PSV) 2 cm proximal to the culprit lesion (‘prestenotic’), within the lesion (‘intrastenotic’), and up to 4 cm distal to the lesion (‘poststenotic’). The ratio of the maximum intrastenotic PSV and the maximum prestenotic PSV (peak velocity ratio; PVR), represented by:


determines the degree of percent stenosis [12]. In-stent restenosis is defined by restenosis >50% and by a peak systolic velocity ratio (PSVR) >2.4 at the lesion site.

### Secondary objectives

The secondary objectives are to assess the efficacy of bare and paclitaxel drug eluting stents in terms of success of the procedure (M1, M12, M24) and quality of life (M1, M12, M24) and to conduct an economic analysis comparing the Zilver® PTX® drug-eluting stent to the Misago® RX bare self-expanding stent.

### Secondary endpoints

The following secondary endpoints will be examined:

- Technical success defined as achievement of a final residual diameter stenosis of <30% on the procedural completion angiogram
- Primary sustained clinical improvement at 1, 12 and 24 months post-procedure defined as a sustained upward shift of 1 category of the Rutherford classification for claudicants and by wound-healing and resting pain resolution for patients in critical limb ischemia, without the need for repeated TLR in surviving patients
- Secondary sustained clinical improvement at 1, 12 and 24 months post-procedure defined as primary sustained clinical improvement including the need for repeated TLR
- Primary patency at 1, 12 and 24 months post-procedure defined as patency without any percutaneous or surgical intervention in the treated segment or adjacent areas
- Major adverse clinical events (MACEs) at 1, 12 and 24 months post-procedure defined as MACEs including all deaths or major amputation
- Limb salvage defined as freedom from major ipsilateral amputations at 1, 12 and 24 months post-procedure
- Death (all causes) at 1, 12 and 24 months post-procedure
- Ankle brachial index at 1, 6, 12 and 24 months post-procedure
- Target extremity revascularization (TER) at 1, 12 and 24 months post-procedure. TER is defined as any percutaneous intervention or surgical bypass of any segment of the target extremity. The target extremity is defined as the ipsilateral limb arteries proximal and distal to the target lesion, including upstream and downstream branches and excluding the target lesion itself
- Target lesion revascularization at 1, 12 and 24 months post-procedure. TLR expresses the frequency of the need for repeated procedures (endovascular or surgical) due to a problem arising from the stent (1 cm proximally and distally to include edge phenomena) in surviving patients with preserved limb
- Stent fracture at 1, 12 and 24 months. Stent fractures, assessed by biplane X-rays. Biplane X-rays, should be performed with two different projections separated by at least 45°, with the patient in a prone position. If this strategy does not cover the entire stented segment, additional views should be obtained. High-resolution images should be obtained either in a diagnostic X-ray room or a fixed unit angiography suite and saved to DICOM files
- Quality of life at 1, 12 and 24 months assessed according the EuroQol-5D-3 L (EQ-5D-3 L) questionnaire
- Economic analysis endpoints: incremental cost-effectiveness ratio based on quality of life for cost-utility analysis and on freedom from in-stent restenosis for cost-effectiveness analysis will be performed to conduct an economic analysis

## Population

### Recruitment

All patients presenting with chronic symptoms of lower extremity peripheral arterial disease will be screened for participation in the clinical investigation. A member of the research team previously trained in the study protocol should screen patients admitted for a percutaneous femoropopliteal artery revascularization procedure for study eligibility. Patients who fail to satisfy inclusion and exclusion criteria will not be randomized in this study. Patients meeting the general inclusion and exclusion criteria will be asked to sign, date and time an informed consent for Switzerland. In France, an informed consent will not be required and information will be given to the patient since the BATTLE trial is considered as a current health care study. After inclusion, a randomization number and treatment arm must be assigned by an interactive web-based randomization service.

### Inclusion criteria

- Patient ≥18 years
- Patient has a history of symptomatic peripheral arterial disease (Rutherford classification: 2 to 5)
- Lesion is eligible for treatment with a maximum of two stents per lesion (treatment of both legs is not permitted)
- *De novo* atherosclerotic lesions (stenosis and/or occlusion) of the superficial femoral artery (SFA), the proximal popliteal artery (P1), or both. The treatment area in the SFA and popliteal artery extended from 1 cm below the origin of the profunda femoral artery to 3 cm above the proximal margin of the intercondylar fossa of the femur
- Resting ankle brachial index (ABI) <0.9
- Patient is affiliated to the Social Security or equivalent system
- Patient has been informed of the nature of the study, agrees to its provisions prior to any study-related procedure
- Patient agrees to undergo all protocol-required follow-up examinations and requirements at the investigational site
- Reference vessel diameter 4 to 7 mm determined by computed tomography (CT) scan (reference vessel diameter obtained from averaging 5-mm segments proximal and distal to the lesions)
- Target lesion has a preprocedure percent diameter stenosis of ≥50% diameter stenosis
- Target lesion has a length ≥2 cm and ≤14 cm
- At least 1 patent runoff vessel (<50% diameter stenosis throughout its course). The inflow artery(ies) cannot be treated using a drug eluting stent or drug coated balloon

### Exclusion criteria

- Asymptomatic lesion
- Restenosis
- No atheromatous disease
- Untreated >50% diameter stenosis of the inflow tract
- Female of childbearing potential
- Patient has received, or is on the waiting list for a major organ transplant
- Patient has a history of coagulopathy or will refuse blood transfusions
- Patient is receiving or scheduled to receive anticancer therapy for malignancy within 1 year prior to or after the procedure
- Severe concomitant disease with life expectation <1 year
- Known allergy to paclitaxel
- Contraindication to aspirin or clopidogrel and ticlopidin (the patient must be able to receive dual antiplatelet treatment for 2 months after the procedure)
- Patient has an infected wound or osteomyelitis on the ipsilateral extremity or foot
- Patient has had prior major amputation to the ipsilateral (target) extremity
- Patient is not able to give informed consent (for the Swiss center only)
- Patient is currently participating in an investigational drug or device study that has not completed the primary endpoint or that clinically interferes with the current study endpoints (Note: trials requiring extended follow-up for products that were investigational, but have become commercially available since then, are not considered investigational trials)
- Patient has previously had, or requires, bypass surgery, endarterectomy or other vascular surgery on any vessel of the ipsilateral extremity
- In the investigator’s opinion, the patient has co-morbid condition(s) that could limit the patient’s ability to participate in the study, compliance with follow-up requirements or impact the scientific integrity of the study
- Target lesion lies within or adjacent to an aneurysm
- Patient with an allergy to contrast agent
- Patient with a severe allergy to metal

### Randomization

Randomization will be conducted via Capture System software by connecting to the website: https://www.dirc-hugo-online.org/csonline/. The connection will be made via a login, password and study name (BATTLE), given by a data manager from the Nantes University Hospital Research Department. The following information should be provided: the first letter of the first name, the first letter of the surname, the birth date, the compliance with inclusion criteria and exclusion criteria (yes/no).

The number and the randomization arm will be assigned automatically at the time of randomization. A statistician, from the Nantes University Hospital Research Department, will prepare the randomization list. An explanatory guide of randomization will be available online via Capture System.

## Interventions

### Description of the investigational devices

The Misago® RX is a peripheral bare metal stent (Misago® RX, Terumo Corp., Tokyo, Japan) indicated to treat iliac and femoropopliteal arteries. The Misago® RX is a flexible self-expanding nitinol stent that is delivered via an RX monorail delivery catheter.

The Zilver® PTX® (Cook Medical, Bloomington, IN, USA) is a peripheral drug eluting stent with a polymer-free paclitaxel coating indicated to treat the above-the-knee femoropopliteal arteries. The anti-proliferative drug is paclitaxel, a cytotoxic drug. The Zilver® PTX® is a flexible self-expanding nitinol stent that is delivered via an over-the-wire system.

### Procedure

Local anesthesia with conscious sedation is recommended unless general anesthesia is required. Access to the culprit SFA lesion is achieved at the investigator’s discretion either by way of a retrograde approach from the contralateral femoral artery with the use of a dedicated 6 F sheath. An intravenous bolus of 50 IU/kg heparin is administered. The same antiplatelet regimen is recommended for all patients: clopidogrel starting at least 24 hours before the intervention or a procedural loading dose of 300 mg orally. Stenotic lesions are crossed in an intraluminal fashion and occlusions are recanalized at the physician’s discretion (intraluminal or subintimal recanalization should be recorded in the electronic case report form; eCRF). A guidewire is positioned through the lesion. Primary stenting is preferably performed using the assigned stent. The stent dimensions are determined according the baseline morphological analysis. The stent diameter is selected such that 4 to 5.5 mm vessels should be treated with a 6 mm stent and that 5.6 to 7 mm vessels should be treated with a 7 mm stent (CT scan estimate); and the length exceeds the lesion length by 2 to 5 mm proximal and distal. Reference vessel diameter is determined by CT scan (reference vessel diameter obtained from averaging 5-mm segments proximal and distal to the lesions). The largest diameter should be used. Stents are placed at least 1 cm below the origin of the profunda femoral artery to 3 cm above the proximal margin of the intercondylar fossa of the femur. A maximum of 10 mm overlap is allowed in cases requiring 2 stents. Preinflation and postinflation are performed at the physician’s discretion. In the event of preinflation or postinflation, the balloon dimension is chosen such that 4 to 5.5 mm vessels should be treated with a 5 mm balloon diameter and that 5.6 to 7 mm vessels should be treated with a 6 mm balloon diameter (CT scan estimate) and so that the balloon length does not exceed that of the stent. Residual diameter stenosis <30% is required for technical success. The technical result of the procedure is assessed by digital subtraction angiography. All associated inflow or outflow lesions suspected to be involved in the disease are treated during the same procedure. Drug eluting devices are forbidden to treat inflow and/or outflow lesions. Groin closure could be accomplished via manual compression or using a vascular closure device. Each enrolling investigator must review the most recently updated instructions for use and assess the contraindications, warnings, potential adverse events and precaution sections for treating potential patients.

## Follow-up (Table 1)

**Table 1:** **Study schedule**

Procedure/Test	Baseline (within 60 days)	Procedure	4 weeks (±2 weeks) office visit	6 months (±1 month)	12 months (±1 month) office visit	24 months (±1 month) office visit	Unschedul d visits
Patient medical/Clinical history	✓						
Patient informed consent (for Swiss centers)	✓						
General inclusion/Exclusion criteria	✓						
Rutherford classification	✓		✓	✓	✓	✓	✓
Quality of life questionnaire	✓		✓		✓	✓	
Preoperative angiography, CT scan or MRA	✓						
Angiographic/Anatomic inclusion/exclusion criteria	✓						
Peripheral angiogram with runoff		✓^1^					
Ankle brachial index (ABI)/Toe brachial index if ABI >1.3 or not able to be reliably measured	✓		✓	✓	✓	✓	
Duplex ultrasound	✓		✓ (within the first 4 weeks)	✓	✓	✓	
Stent radiography			✓		✓	✓	
Per protocol medications^2^		✓	✓	✓	✓	✓	✓
Concomitant medications	✓	✓	✓	✓	✓	✓	✓
Adverse events/Device deficiencies/Adverse product experiences		✓	✓	✓	✓	✓	✓

^1^Peripheral angiogram (procedural/post-procedure) for all patients.

^2^Aspirin ≥75 mg daily must be given for a minimum of 2 months, and clopidogrel 75 mg daily to be taken throughout the length of the study (2 years) post-procedure.

CT, computed tomography.

MRA, magnetic resonance angiogram.

### Follow-up schedule

All patients randomized into the clinical investigation will have clinical follow-up at:

- 4 weeks post-procedure ±2 weeks
- 12 months post-procedure ±1 month
- 24 months post-procedure ±1 month

All patients will undergo quality of life assessment at:

- 4 weeks post-procedure ±2 weeks
- 12 months post-procedure ±1 month
- 24 months post-procedure ±1 month

All patients will undergo a duplex scan at:

- Within the first 4 weeks
- 6 months post-procedure ±1 month
- 12 months post-procedure ±1 month
- 24 months post-procedure ±1 month

All patients will undergo stent radiography at:

- 4 weeks post-enrolment ±2 weeks
- 12 months post-procedure ±1 month
- 24 months post-procedure ±1 month

### Follow-up medications

All patients randomized into the study will be maintained on 2 antiplatelet agents combining aspirin (75 mg) and a thienopyridine. Aspirin (75 mg) will be given daily for a minimum of 2 months following the procedure. A thienopyridine (for example, 75 mg of clopidogrel) must be taken throughout the length of the study (2 years). If a patient develops sensitivity to clopidogrel, they may be switched to ticlopidine hydrochloride at a dose according to standard hospital practice. When patients are receiving oral anti-coagulant treatment, aspirin is the only antiplatelet agent added. These medications can be halted for medical necessity if required. However, they must be resumed as soon as possible per physician discretion. The start of antiplatelet medications and termination of will be documented in the eCRF. The medication history should be updated as needed to include modifications to the concomitant medications and protocol-required medications.

### Duplex scan follow-up

Patients will have a follow-up by duplex scan within the first 4 weeks, 6 months (±1 month), 12 months (±1 month) and 24 months (±1 month). Doppler ultrasound examinations will be registered on a CD or DVD. Information will be collected on standardized forms filled during duplex scan examination including Rutherford classification, ABI, PSVR, run-off, per-protocol medications and adverse events. The forms and the CD (or DVD) will be returned to the investigator. High-resolution images should be saved. Duplex scan examinations will be downloaded in the eCRF to be analyzed online by an independent core laboratory.

### Stent radiography follow-up

To standardize evaluation of stent fractures, biplane X-rays of the femoropopliteal arterial segment (including the hip and the knee) should be performed to cover the entire stented segment. An exposure <80kVp using a magnification of 1.5 image intensifier/receptor magnification is recommended ato obtain the greatest coverage of the stent. If this strategy is insufficient, additional views should be obtained. X-rays should be performed with 2 different projections separated by at least 45°, with the patient in a prone position. High-resolution images should be saved to DICOM files. Stent radiography will be downloaded in the eCRF to be analyzed online by an independent core laboratory.

### Type of comparison

The study objective is to determine whether DES (Zilver® PTX®) will be superior to a latest generation bare nitinol stent (Misago® RX, Terumo Corp., Tokyo, Japan) in the treatment of intermediate length femoropopliteal lesions in patients with symptomatic peripheral arterial disease (Rutherford 2 to 5).

### Type of analysis

According to the intent to treat principle, all randomized patients will be included in this analysis and a censoring mechanism will be applied to those patients without an event over 1 year of study follow-up. The time-to-first event (in-stent restenosis) will be calculated as the first restenosis date. Patients without an in-stent restenosis at the end of 1 year of study follow-up will have their efficacy measure censored at M12. Patients who withdraw from the study before completing 1 year of study follow-up, and have not experienced an event, will have their time-to-event measure censored on their withdrawal date. Patients without an event and who are lost to follow-up will be censored at the day of last contact. This concept will be applied to both Zilver® PTX® and Misago® patients. Every effort will be made to have zero patients lost to follow-up and to encourage the investigator to keep patients under study observation. In both arm groups, for patients who died before the final follow-up examination or for patients lost to follow-up, the status of the last follow-up examination was recorded.

### Power calculation

The null (H0) and alternative (HA) hypotheses for the primary endpoint of freedom from in-stent restenosis at 1 year are:

H0: freedom from in-stent restenosis at 1 year (Zilver® PTX®) ≠ freedom from in-stent restenosis at 1 year (Misago® RX)

HA: freedom from in-stent restenosis at 1 year (Zilver® PTX®) = freedom from in-stent restenosis at 1 year (Misago® RX)

The sample size calculation for the primary endpoint is based on the following assumptions: two-sided superiority test, α =0.05, randomization ratio is 1 (Zilver® PTX®): 1 (Misago® RX arm), and a power of 80%. The true freedom from in-stent restenosis rate is assumed to be 86.2% for Zilver® PTX® arm [8]. The BATTLE trial is designed to assess primary stenting in the treatment of intermediate length femoropopliteal lesions. Consequently, we have used Vienna, Astron and Durability results in which the mean length of the treated lesions is longer (101, 82 and 96.4 mm respectively). Indeed, in the Misago trial the 750 femoropopliteal lesions were treated with a mean lesion length of 63 mm. Therefore, the average of the true freedom from in-stent restenosis rate in the bare metal stent group was 66.9% (Δ for superiority = 0.862 to 0.669 = 19.3%) [2–4]. S-PLUS software (TIBCO, CA, USA) will be used to determine the appropriate sample size for detecting the difference between two proportions. Based on the above assumptions, a total of 170 patients (85 patients for each arm) will provide approximately 80% power. Assuming an approximately 10% dropout rate at 1 year, 186 patients will be randomized (93 patients for each arm). The variance needed to construct the test statistic depends on the parameters being tested. It seems reasonable to use all of the data available to estimate the variances, and this is exactly what S-PLUS does. A weighted average of the two estimates for the proportions will be used to estimate the variance under H0.

### Statistical analysis

The primary statistical methodology for this study will be based on the Kaplan-Meier estimates of the survivor function, life table estimates of the survivor and hazard ratio (95% CI) computed using the Cox proportional hazards model. A log rank test will be used to assess the statistical significance of observed arm differences in the time-to-event distributions between the Zilver® PTX® and Misago® groups. The log rank test statistics, *P*-values, Kaplan-Meier estimates, and life table estimates will be obtained from the SAS® V9.3 procedure LIFETEST (Cary, NC, USA). The Cox proportional hazards model will be used to obtain an estimate of the hazard ratio for the Zilver® PTX® group to the Misago® group. A 95% confidence interval will be computed for the hazard ratio. In addition, the Cox proportional hazards model with group and potential baseline variables will be used to estimate the adjusted hazard ratio of Zilver® PTX® group to the Misago® RX group. Analysis of the primary endpoint will be on a per-patient basis. Analysis based on the per treatment evaluable population will also be performed.

### Trial management and quality assurance

A clinical research assistant (CRA) representing the sponsor will schedule monitoring visits regularly. During these visits, the CRA will review study plan compliance, adherence to the protocol, and data quality. The CRA will compare eCRFs and ensure that the study is being conducted in compliance with pertinent regulatory requirements. The investigator will provide the CRA with direct access to eCRFs and to the subject’s records (for example, medical records, office charts, hospital charts, and study-related charts) for source data verification, as well as any other study documents.

## Discussion

The aim of the BATTLE trial is to compare the paclitaxel eluting stent (Zilver® PTX®) with a latest generation bare self-expendable nitinol stent (Misago® RX, Terumo Corp., Tokyo, Japan) in the treatment of intermediate length femoropopliteal lesions (≤14 cm). We decided to evaluate only intermediate length femoropopliteal lesions for several reasons. Firstly, most trials have investigated femoropopliteal lesions ≤15 cm length with lesion length ranged from 45 to 101 mm and consequently, according to the TASC, endovascular treatment for femoropopliteal lesions is indicated for TASC A and B lesions [1]. Secondly, Zilver® PTX® DES is indicated for lesions ≤14 cm according the inclusion criteria and the results of the Zilver® PTX® trial [8]. Also, the use of DES for longer lesions has not been sufficiently evaluated and, moreover, the maximal length of Zilver® PTX® is 12 cm. Finally, we decided to exclude short lesions (<2 cm) since in the FAST trial, the primary stenting of short femoropopliteal lesions did not show better results than angioplasty.

Our primary endpoint is freedom from in-stent restenosis at 1 year, as assessed by duplex scan. In-stent restenosis is defined by restenosis >50% and by a PVR >2.4 at the lesion site. Duplex scan is a standard clinical technique used to evaluated in-stent stenosis. Currently, duplex scan is the routine modality for imaging follow-up of lower limbs since it is non-invasive, low-cost and the diagnosis value of duplex scan seems comparable to digital subtraction arteriogram [13, 14].

Only *de novo* atheromatous femoropopliteal lesions will be included and, consequently, we have excluded in-stent restenosis. Indeed, the treatment of in-stent restenosis seems different since its composition associates not only an atheromatous plaque, but also a proliferation and migration of smooth muscle cells, an inflammatory process and a matrix deposition and accumulation.

Regarding the antiplatelet regimen, there is no evidence-based medicine in the setting of peripheral arterial disease stenting. Consequently, we have chosen the same protocol as the Zilver® PTX® trial that combines aspirin and clopidogrel for at least 60 days after the procedure and lifelong aspirin therapy [8].

The sample size calculation for the primary endpoint is based on the assumptions of 13.8% and 33.1% of in-stent restenosis in the Zilver® PTX® group and the bare metal stent group respectively. The rate of in-stent restenosis for the DES group was given by the Zilver® PTX® study, whereas the rate of in-stent restenosis for the bare metal stent group was given by the mean of in-stent restenosis of three different studies: Vienna, Astron and Durability [2–4]. In these trials, the mean lesion length and in-stent restenosis rates were 101, 82, 96.4 mm and 37%, 34.4% and 27.8% respectively. Considering these studies, the average in-stent restenosis rate was 33.1%. We considered that these studies were more relevant due the intermediate length of the treated lesions.

One limitation could almost already be addressed: the BATTLE trial is not a blinded study. Indeed, some differences exist according to the device. For example, the Zilver® PTX® stent is delivered via an over-the-wire-delivered catheter, whereas the Misago® RX is delivered via a RX-delivered catheter. Moreover, Zilver® PTX® stent deployment is controlled by retraction of the handle while holding the metal cannula stationary. For the Misago RX®, a small ergonomic handle controls the release of the stent using a single hand.

## Trial status

Recruitment began in February 2014 and is expected to take 2 years.

## Abbreviations

ABI: ankle brachial index
CRA: clinical research assistant
CT: computed tomography
DES: drug eluting stents
eCRF: electronic case report form
EQ: EuroQol
MACE: major adverse clinical event
MRA: magnetic resonance angiogram
PSV: peak systolic velocity
PSVR: peak systolic velocity ratio
PVR: peak velocity ratio
RVD: reference vessel diameter
RX: rapid-exchange
SFA: superficial femoral artery
TASC: Trans-Atlantic Inter-society Consensus Document on Management of Peripheral Arterial Disease
TER: target extremity revascularization
TLR: target lesion revascularization.
